# Relationship Between Gallbladder Wall Thickness and Perioperative Outcomes in Patients Undergoing Laparoscopic Cholecystectomy

**DOI:** 10.7759/cureus.116929

**Published:** 2026-09-25

**Authors:** Jay P Patel, Mohit K Badgurjar, Mitesh Asari, Suman Parihar, Rakshita Saini, Isha Purohit, Hardik V Chaudhary

**Affiliations:** 1 Department of General Surgery, Geetanjali Medical College and Hospital, Udaipur, IND

**Keywords:** cholelithiasis, gallbladder wall thickening, laparoscopic cholecystectomy, perioperative outcomes, ultrasonography

## Abstract

Introduction

Gallbladder wall thickness is a preoperative imaging parameter. Increased gallbladder wall thickness has been associated with difficult organ dissection, which may influence the postoperative outcome of a patient. The present study aims to understand the relationship between gallbladder wall thickness and intraoperative and postoperative outcomes in patients undergoing laparoscopic cholecystectomy.

Materials and methods

This was a prospective comparative study conducted in the Department of General Surgery and Gastrointestinal Surgery over 18 months. The study began after receiving approval from the institute’s ethics committee. Patients with symptomatic cholelithiasis were evaluated preoperatively using ultrasonography to measure gallbladder wall thickness and were subsequently classified into two groups based on their gallbladder wall thickness: Group A (<3 mm) and Group B (≥3 mm). All patients underwent laparoscopic cholecystectomy. Their intraoperative parameters were assessed using an intraoperative difficulty score. Categorical variables between the two groups were compared using the chi-square test and Fisher’s exact test where expected cell counts were less, and continuous variables were compared using an unpaired t-test when required.

Results

After applying the eligibility criteria, a total of 116 patients were enrolled in the study. Group A (<3 mm) comprised 79 patients (68.10%), and Group B (≥3 mm) comprised 37 patients (31.90%). Age (p=0.28) and gender distribution (p=0.22) showed no statistical significance. Symptom presentation, such as fever, vomiting, and pain radiating to the back, differed significantly between the two groups (p<0.001). Intraoperative difficulty scoring showed that 82.28% (n=65) of participants in Group A were classified as having an easy procedure, followed by a relatively difficult procedure (n=13, 16.46%) and a difficult procedure (n=1, 1.27%). In Group B, a majority had a difficult procedure (n=16, 43.34%), followed by easy procedures (n=12, 32.43%) and relatively difficult procedures (n=9, 24.32%). This difference was statistically significant (p<0.001). Similarly, time to return of bowel sounds was shorter in Group A than in Group B (3.53 ± 2.04 vs 4.93 ± 1.62, p < 0.001), and the duration of hospital stay also differed significantly between the two groups (1.72 ± 0.58 vs 2.54 ± 0.90 days; p<0.001).

Conclusion

Preoperative gallbladder wall thickness is an important ultrasonography parameter that can be used to assess the intraoperative and postoperative outcomes in patients undergoing laparoscopic cholecystectomy.

## Introduction

With an estimated prevalence of 6.1%, cholelithiasis is one of the most common disorders of the hepatobiliary system, representing a major cause of morbidity worldwide [1]. For symptomatic cholelithiasis, laparoscopic cholecystectomy is the standard treatment [2].

Gallbladder wall thickness is defined as the distance between the inner mucosal surface and the outer serosal surface of the gallbladder. A wall thickness less than 3 mm is generally considered normal, while values exceeding this limit are regarded as abnormal [3,4]. Increased wall thickness has been associated with distorted anatomy, dense adhesions, and friable tissues [5]. Such factors are known to complicate the surgical dissection of the organ by making it difficult to identify critical anatomical landmarks, increasing the risk of intraoperative bleeding, and injury to adjacent structures [5,6]. Such intraoperative challenges may have a direct impact on postoperative outcomes, including the need for drain placement, postoperative pain, longer hospital stay, and higher complication rates.

Ultrasonography is the primary imaging modality for evaluating gallbladder disease and assessing gallbladder wall thickness [7]. Accurate ultrasonographic measurement of gallbladder wall thickness, particularly when performed under standardized conditions, provides valuable insight into gallbladder inflammation and aids preoperative risk assessment [7]. Therefore, gallbladder wall thickness is increasingly recognized as a potential predictor of operative difficulty and adverse surgical outcomes.

Thus, evaluating the relationship between gallbladder wall thickness and perioperative outcomes such as intraoperative and postoperative outcomes is clinically important. The primary objective of this study was to evaluate the association between gallbladder wall thickness and intraoperative difficulty. The secondary objective of this study was to assess the association between gallbladder wall thickness and postoperative outcomes such as postoperative day of discharge and time to appearance of bowel sounds in patients undergoing laparoscopic cholecystectomy at a tertiary care centre in South Rajasthan.

## Materials and methods

Study setting

This study was designed as a prospective comparative observational study. It was conducted in the Department of General Surgery and Gastrointestinal Surgery at Geetanjali Medical College and Hospital over 18 months, from 29th April 2024 to 29th October 2025. The study aimed to evaluate the relationship between gallbladder wall thickness and perioperative outcomes in patients undergoing laparoscopic cholecystectomy to predict difficult gallbladder surgery preoperatively. The study was carried out after obtaining approval from the Institutional Human Research Ethics Committee (HREC) and Institutional Research Review Committee (IRRC) with approval number GU/HREC/EC/2024/2460. Informed consent was obtained from all the participants before enrollment in the study. 

Patient selection and eligibility criteria

The present study enrolled all of the patients who fulfilled the eligibility criteria and presented to the Outpatient Department of General Surgery and Gastrointestinal Surgery with complaints and findings suggestive of symptomatic cholelithiasis. The eligibility criteria are presented in Table 1. 

**Table 1:** Inclusion and Exclusion Criteria for Patient Enrollment

Inclusion Criteria	Exclusion Criteria
Age more than 18 years	Failure to give informed consent
Ultrasonographically confirmed cases of cholelithiasis and variable presentation of cholecystitis	Patients with obstructive biliary pathology
	Pregnancy
	Patients unfit for general anesthesia

Sample size calculation

The participants were recruited using convenience sampling, which enrolled all patients who presented to the Department of General Surgery and Gastrointestinal Surgery during the study period and fulfilled the eligibility criteria. 

Study design

All patients underwent detailed clinical evaluation, including history taking and physical examination using a pre-designed proforma. Routine clinical investigations were done, along with liver function tests, and all patients underwent transabdominal ultrasonography in the fasting state as part of the standard preoperative workup. Magnetic resonance cholangiopancreatography (MRCP) was performed selectively in patients with deranged liver function. Ultrasonographic evaluation was performed using a curvilinear transducer (2-5 MHz) after a minimum fasting period of six hours. The gallbladder was examined in multiple longitudinal and transverse planes with the patient in supine and left lateral decubitus positions. Gallbladder wall thickness was measured at the anterior wall, perpendicular to the wall, at its thickest point while avoiding tangential measurements. The presence of gallstones or sludge, gallbladder distension, pericholecystic fluid, wall stratification, hyperemia on color Doppler, and sonographic Murphy sign was also documented. Gallbladder wall thickness was measured as the distance between the inner mucosal and outer serosal surfaces. Patients were assigned to Group A (gallbladder wall thickness <3 mm) or Group B (gallbladder wall thickness ≥3 mm) based on this measurement. The procedure was performed by a single radiologist in order to minimize interobserver variability. All patients underwent laparoscopic cholecystectomy under standard operative protocols. Intraoperative findings were recorded, including operative time, presence and type of adhesions, intraoperative bleeding, bile spillage, need for drain placement, and conversion to open cholecystectomy. 

Intraoperative difficulty grading 

An intraoperative difficulty score for this study was adapted from the existing scoring system [8,9]. It assessed six parameters: operative duration, adhesions, bleeding, need for drain placement, conversion to open surgery, and bile spillage. Four parameters (operative duration, adhesions, bleeding, and bile spillage) contributed 0-2 points, while two parameters (need for drain placement and conversion to open surgery) contributed 0-1 point, with a total range of 0-10. Higher scores denoted easier operation. The operational definition of operative duration and intraoperative blood loss were derived from institutional experience along with previously reported values of difficult laparoscopic cholecystectomy [10]. Total scores were classified as easy (>6), relatively difficult (4-6), or difficult (≤3). The content validation of the intraoperative difficulty score was carried out by experts who assessed each item for relevance and clarity, yielding a content validity index of 0.84. Pilot testing for the score was done among 10 patients whose data were not included in the final analysis. The intraoperative difficulty scoring system is presented in Table 2. 

**Table 2:** The Intraoperative Difficulty Scoring System Total score range: 0 to 10. Score interpretation: >6=easy; 4-6=relatively difficult; ≤3=difficult. A higher score denotes a technically easier operation.

Intraoperative Parameter	2 points	1 point	0 points
Operative duration	≤2 hours	>2 to 3 hours	>3 hours
Adhesions	None	Thin/flabby	Dense
Bleeding	Minimal (<50 mL)	Mild (50-100 mL)	Moderate (>100 mL)
Need for drain placement	—	No	Yes
Conversion to open surgery	—	No	Yes
Bile spillage	None/minimal	Mild	Gross

Postoperative outcomes were evaluated in terms of postoperative day of discharge and the time to appearance of bowel sounds. Patients were discharged when they fulfilled three criteria: Vitally stable, passed flatus/ feces, and tolerated oral intake with no surgical complaints requiring hospital stay. Bowel sounds were auscultated over the right lower quadrant, and their return was defined as the presence of 3 to 5 sounds per minute, auscultated over three minutes. 

Statistical analysis 

Data were collected and analyzed using Microsoft® Excel for Mac, version 16.110.3 (26070318), licensed under Office Home 2024 (Microsoft Corporation, Redmond, WA, USA) and IBM SPSS Statistics for Windows, Version 21 (Released 2012; IBM Corp., Armonk, New York, United States). Categorical variables were expressed as frequencies and percentages and compared using the chi-square test and Fisher’s exact test where expected cell counts were less than 5. Continuous variables were expressed as mean ± standard deviation and evaluated using an unpaired t-test. A p-value <0.05 was considered statistically significant.

## Results

Gallbladder wall thickness

A total of 116 patients were included in the study. Based on preoperative gallbladder wall thickness assessment, 79 patients (68.10%) were allocated to Group A (gallbladder wall thickness <3 mm), while 37 patients (31.90%) were allocated to Group B (gallbladder wall thickness ≥3 mm). Thus, the majority of the study population belonged to Group A, indicating that most patients undergoing laparoscopic cholecystectomy had a gallbladder wall thickness of <3 mm on preoperative ultrasonography. The mean stone size in Group A was 8.70 ± 3.87 mm, and 12.17 ± 4.20 in Group B, with the difference being statistically significant (p<0.001). The data are presented in Table 3. 

**Table 3:** Distribution of Gallbladder Wall Thickness and Mean Stone Size Among Study Groups SD: Standard Deviation

Characteristics	Group A (<3 mm)	Group B (≥3 mm)	t-value	p-value
Gallbladder wall thickness, n (%)	79 (68.10%)	37 (31.90%)	-	-
Mean stone size, mm (mean ± SD)	8.70 ± 3.87	12.17 ± 4.20	4.38	<0.001

Demographic variables

The mean age in Group A was 50.39 ± 12.67 years and 52.83 ± 10.83 years in Group B, and the difference was not statistically significant (p=0.28). The gender distribution showed a female predominance in both study groups (Group A: n=50, 63.29%; Group B: n=19, 51.35%). Statistical analysis using the chi-square (χ²) test showed no significant difference in gender distribution. The data are presented in Table 4. 

**Table 4:** Age and Gender Distribution of Study Participants in Group A (<3 mm) and Group B (≥ 3 mm) #Differences in age and gender between Group A (<3 mm) and Group B (≥ 3 mm) were calculated using the unpaired t-test and chi-square (χ²) test.

Characteristics	Group A, n (%)	Group B, n (%)	Test-statistic^#^	p-value
Age, mean ± SD	50.39 ± 12.67	52.83 ± 10.83	t-test= -1.07	0.28
Male, n (%)	29 (36.71)	18 (48.65)	χ² value=1.49	0.22
Female, n (%)	50 (63.29)	19 (51.35)		

Clinical characteristics

In both groups, a majority of patients presented with more than one symptom. Upper abdominal pain was the most common symptom reported across both groups (n=62, 78.48% in Group A and n=34, 91.89% in Group B); however, the difference was not statistically significant (p=0.075). The distribution of presenting symptoms such as pain radiating to the back, vomiting, and fever showed a statistically significant difference between the two groups (p<0.001), and postprandial fullness also showed a statistically significant difference between the two groups (p<0.011). The data are presented in Table 5.

**Table 5:** Distribution of Symptoms Among Participants in Group A (<3 mm) and Group B (≥ 3 mm) #Differences in symptoms between Group A (<3 mm) and Group B (≥ 3 mm) were calculated using the chi-square (χ²) test and Fisher’s exact test where expected cell counts were low.

Symptoms	Group A, n (%)	Group B, n (%)	Test-statistic^#^	p-value
Pain radiating to back	1 (1.27)	11 (29.73)	Fisher’s exact	<0.001
Vomiting	1 (1.27)	19 (51.35)	Fisher’s exact	<0.001
Fever	0 (0)	7 (18.92)	Fisher’s exact	<0.001
Postprandial fullness	53 (67.09)	33 (89.19)	χ² value=6.42	0.011
Upper abdominal pain	62 (78.48)	34 (91.89)	χ² value=3.18	0.075

Intraoperative difficulty score

The overall intraoperative difficulty score differed significantly between the two groups (p<0.001). In Group A, the majority of patients (82.28%, n=65) had an easy procedure (score>6), followed by relatively difficult procedures (n=13, 16.46%). In contrast, Group B demonstrated a substantially higher proportion of difficult procedures, observed in 43.24% (n=16) of patients, followed by an easy procedure (n=12, 32.43%). The difference between the two groups differed significantly between the two groups (p<0.001). The detailed distribution of operative duration, adhesions, bleeding, drain placement, conversion to open surgery, and bile spillage are presented in Table 6 and the composite score is presented in Table 7. 

**Table 6:** Comparison of Intraoperative Findings between Group A (<3 mm) and Group B (≥3 mm)

Variable	Category	Group A (n = 79), n (%)	Group B (n = 37), n (%)
Operative duration	≤2 hours	62 (78.48%)	11 (29.73%)
	>2 to 3 hours	16 (20.25%)	14 (37.84%)
	>3 hours	1 (1.27%)	12 (32.43%)
Adhesions	None	39 (49.37%)	7 (18.92%)
	Thin/flabby	35 (44.30%)	16 (43.24%)
	Dense	5 (6.33%)	14 (37.84%)
Bleeding	Minimal (<50 mL)	70 (88.61%)	16 (43.24%)
	Mild (50–100 mL)	3 (3.80%)	8 (21.62%)
	Moderate (>100 mL)	6 (7.59%)	13 (35.14%)
Drain placement	No	79 (100.00%)	35 (94.59%)
	Yes	0 (0.00%)	2 (5.41%)
Conversion to open surgery	No	79 (100.00%)	37 (100.00%)
	Yes	0 (0.00%)	0 (0.00%)
Bile spillage	None/minimal	48 (60.76%)	16 (43.24%)
	Mild	30 (37.97%)	12 (32.43%)
	Gross	1 (1.27%)	9 (24.32%)

**Table 7:** Comparison of the Overall Intraoperative Difficulty Score Between Group A (<3 mm) and Group B (≥ 3 mm) Differences in overall intraoperative difficulty score between Group A (<3 mm) and Group B (≥ 3 mm) were calculated using Fisher’s exact test.

Groups	Easy (>6), n (%)	Relatively Difficult (4-6), n (%)	Difficult (≤3), n (%)	p-value
Group A	65 (82.28)	13 (16.46)	1 (1.27)	<0.001
Group B	12 (32.43)	9 (24.32)	16 (43.24)	

Postoperative findings

The mean time to appearance of bowel sounds was 3.53 ± 2.04 hours in Group A and 4.93 ± 1.62 hours in Group B, which differed significantly between the groups (p<0.001). The postoperative day of discharge was 1.72 ± 0.58 days in Group A and 2.54 ± 0.90 days in Group B, which also differed significantly (p<0.001). The data are presented in Table 8.

**Table 8:** Comparison of Gallbladder Wall Thickness and Postoperative Parameters in Group A (<3 mm) and Group B (≥3 mm) #Differences in postoperative time to appearance of bowel sounds between Group A (<3 mm) and Group B (≥3 mm) were calculated using an unpaired t-test. SD: Standard deviation

Postoperative Parameters	Group A	Group B	Test Statistic^#^	p-value
Time to appearance of bowel sounds, hours, (mean ± SD)	3.53 ± 2.04	4.93 ± 1.62	t-test= -3.98	<0.001
Postoperative day of discharge (mean ± SD)	1.72 ± 0.58	2.54 ± 0.90	t-test= -5.071	<0.001

## Discussion

The goal of the current prospective comparative observational study was to assess the association between gallbladder wall thickness and perioperative outcomes such as intraoperative and postoperative outcomes in patients undergoing laparoscopic cholecystectomy. Based on preoperative ultrasonographic measurements of gallbladder wall thickness, 116 patients with symptomatic cholelithiasis were included and split into two groups, Group A (gallbladder wall thickness <3 mm, n=79, 68.10%) and Group B (gallbladder wall thickness ≥3 mm, n=37, 31.90%). There was a female predominance in Group A with a female-to-male ratio of 1.72:1, and 1.06:1 in Group B; however, there was no significant difference between the two groups. These findings correlate with the epidemiological patterns of gallstone disease, where higher female dominance is seen. This is attributed to the estrogen-induced increase in cholesterol saturation of bile [11]. Khan et al. also reported similar demographic profiles in their study, with female predominance among patients undergoing laparoscopic cholecystectomy for symptomatic gallstone disease [4]. In the literature, the male sex has been associated with more severe gallbladder inflammation, which may lead to difficult cholecystectomy [12,13]. Although the percentage of male patients in Group B (48.65%) was numerically higher than in Group A (36.71%), no statistically significant difference was found.

The distribution of several presenting symptoms, such as pain radiating to the back, fever, and vomiting, showed statistical significance between the two groups (p<0.001, respectively), along with postprandial fullness (p=0.011). The presence of higher frequency of symptoms suggested that the patients with thickened gallbladder walls presented with advanced gallbladder disease. This further aligns with findings from Ganesh et al., who reported that patients with severe gallbladder wall thickening experienced a higher incidence of prolonged ileus and other postoperative complications, suggesting that severe thickening was associated with a more complex clinical presentation and recovery course [14].

The distribution of gallbladder wall thickness showed that 68.10% of patients had gallbladder wall thickness <3 mm (Group A) and 31.90% had gallbladder wall thickness ≥3 mm (Group B). Group B had a higher mean stone size (12.17 ± 4.20 mm) than Group A, which was statistically significant (p<0.001). This is consistent with Alotaibi, who observed greater stone sizes in patients with higher gallbladder wall thickness, suggesting that larger gallstones are more likely to be impacted at the neck, thus causing prominent inflammatory changes, leading to gallbladder wall thickening [15].

Our study demonstrated an association between gallbladder wall thickness and intraoperative difficulty scoring during laparoscopic cholecystectomy, which was also statistically significant (p<0.001). In Group A (<3 mm), 82.28% (n=65) of patients experienced an easy operation (score>6), while in Group B, 43.24% (n=16) of patients had a higher proportion of difficult operations (score≤3), suggesting that patients with thickened gallbladder walls were associated with more technically demanding procedures.

Our results align closely with the findings of several previous studies that have evaluated the predictive value of gallbladder wall thickness for surgical difficulty. Chindarkar et al. reported that only 15.2% of patients with thinner walls had difficult surgery, compared to 92.9% of patients with gallbladder wall thickness >4 mm (p<0.001) [16]. Similarly, Bansal et al. observed that only 1.3% of patients with thinner walls had surgical difficulties, whereas 43.6% of patients with gallbladder wall thickness >4 mm did (p<0.001) [17]. Durmus et al. support this by demonstrating that gallbladder wall thickness ≥4 mm was significantly associated with increased surgical difficulty (p<0.001) and that the risk of conversion to open surgery was 15.68 times higher in the difficult group [18]. Zhu et al. further strengthened this evidence by identifying gallbladder wall thickness as an independent predictor of difficult laparoscopic cholecystectomy (p=0.0007) [19]. Bahadorzadeh et al. also found that gallbladder wall thickness increased the odds of difficult surgery by 2.9 times [20]. This highlights the importance of preoperative gallbladder wall thickness and its impact on perioperative outcomes. 

There was a significant association between gallbladder wall thickness and the time it took for postoperative time to appearance of bowel sounds to occur. In our study, patients in Group A had significantly earlier return of bowel function compared to Group B (p<0.001). This finding can be explained by several possible mechanisms, such as longer operating times leading to increased intestinal loop manipulation (especially when the duodenum and colon are adhered to the gallbladder), which may result in postoperative ileus [21]. Furthermore, Ganesh et al. reported that patients with severe gallbladder wall thickening had a higher incidence of prolonged ileus, which is consistent with our findings of delayed return of bowel movement [14].

In Group B, the postoperative hospital stay was substantially longer than in Group A (p<0.001). In Group A, approximately 96.20% of patients (n=76) were discharged on postoperative day 1 and 2; on the other hand, 43.24% (n=16) were discharged on postoperative day 3 and 4 in Group B. These results align with those of Khan et al., who reported that individuals with moderate and severe gallbladder wall thickening had considerably longer hospital stays following surgery than patients with normal wall thickness [4]. 

Raman et al. demonstrated that patients with greater gallbladder wall thickness had progressively longer hospital stays, increasing from 1.09±1.42 days in those with normal wall thickness to 3.54±4.61 days in those with severe wall thickening (p<0.001). Additionally, increasing wall thickness was associated with higher rates of conversion to open surgery and postoperative complications, suggesting that severe gallbladder wall thickening is an important predictor of adverse surgical outcomes [5].

It is important to note that “difficult cholecystectomy” is a subjective term and may vary from surgeon to surgeon depending on their experience and expertise. However, within the limits of this study, a gallbladder wall thickness of more than 3 mm may provide an objective idea of intraoperative difficulty. This parameter may be useful in the early operative experience of a surgeon by serving as a preoperative indicator of a difficult procedure. This may be particularly useful in peripheral healthcare settings where access to surgical, radiological, and endoscopic facilities is limited, thus allowing them to make necessary arrangements such as senior assistance or possible referral to a higher center with adequate facilities. 

Limitations

There are a few limitations to this study. First, the relatively small number of patients included in the sample (n=116), along with unequal distribution of participants between the two groups, may limit the statistical power and generalizability of the findings to other patient samples. Second, this was a single-center study, thus limiting the external applicability of the results. Third, the use of convenience sampling may introduce selection bias in the study. Fourth, the influence of gallbladder wall thickness on postoperative complications such as bile duct injury was not examined due to the required larger number of patients for such an analysis. Fifth, due to the lack of multivariable analysis, all associations reported are unadjusted and unaccounted for. Another limitation was that the intraoperative difficulty scoring used in our study underwent pilot testing and content validation within our institute and has not been externally validated. Additionally, currently there exists no standard cutoff for gallbladder wall thickness, with the value differing in the aforementioned studies. The 3 mm cutoff used in our study was pre-specified, and our findings should be interpreted accordingly. 

## Conclusions

This study has shown that thickening of the gallbladder wall is associated with poor intraoperative and postoperative results in patients undergoing laparoscopic cholecystectomy for symptomatic cholelithiasis. A large proportion of patients with thickened gallbladders had difficult cholecystectomy. These findings emphasize that preoperative measurement of gallbladder wall thickness by ultrasonography can serve as one of the important prognostic markers for surgery. Understanding the role of gallbladder wall thickness in predicting the operative outcome will help surgeons prepare themselves for difficult cases.
